# Influence of open-top chambers induced climate warming on secondary metabolic profile of culturally and medicinally important plants of Himalaya, Karakoram and Hindukush

**DOI:** 10.1371/journal.pone.0322480

**Published:** 2025-05-14

**Authors:** Saira Karimi, Muhammad Ali Nawaz, Saadia Naseem, Zahid Ali

**Affiliations:** 1 Department of Biosciences, Plant Biotechnology & Molecular Pharming Lab, COMSATS University Islamabad (CUI), Islamabad, Pakistan; 2 Research Cell, Bahria University, Health Sciences Campus BUHSCI, Islamabad, Pakistan; 3 Department of Biological and Environmental Sciences, Qatar University, Doha, Qatar.; University of Education, PAKISTAN

## Abstract

Plants native to colder climates, higher elevations, or semi-arid regions have more phenolic compounds in their organs. Faced with the current climate crisis, the effects of global heating with overgrazing pressure on natural pastures are not fully recognized in the Himalaya-Karakoram and Hindukush (HKH) region. The objective of this research was to investigate how biological active compound accumulation and concentration of high-elevation plants change under the influence of simulated climate warming which was induced by open-top chambers. The bioactive profiling plant species from experimental units were investigated through High-Performance Liquid Chromatography (HPLC), and compared with control. That revealed a significant increase in the major phenolic acid and flavonoid compounds (Rutin, Quercetin, Myricetin, Gallic Acid, and Kaempferol). The accumulation of other minor compounds, such as Vanillic Acid, Syringic Acid, Ferulic Acid, and Catechin, showed species-specific variation in concentration. The species-specific responses indicated the dominant and positive adaptation species such as *P. macrophylla, A. rupestris, A. penduncularis, P. hololeuca, P. alpina*, and under stress could accumulate more secondary metabolites, explaining their capacity for adaptation. These species’ proliferation under a stressed climate and higher elevation with grazing pressure provides insightful information about their exploitation of phenolic compounds which may alter the environmental sustainability.

## Introduction

Metabolomics is a field that explores the effects of environmental factors on plant bioactive compounds. The accumulation of metabolites in the plant species infinitely depends on different environmental conditions. It explains that the respective group of secondary metabolites acts as a chemical boundary between the plant and its environment [1,2]. The biological role of secondary metabolites are to generate adaptive responses to the changes in the surrounding environment [3,4]. These responses include the changes in the synthesis and accumulation of plant secondary metabolites[5]. The variation in metabolite levels of different medicinal plant species growing under climate change stress plays a crucial role when determining the chemical characterization of these plants [6]. Key factors contributing to these variations may include acclimation, the onset of phenological responses, and other region-specific biotic and abiotic factors, [7–12]. External environmental factors such as temperature, rainfall, light, water, and nutrient properties affect the synthesis and concentration of secondary metabolites. The experts have warned that climate challenges would lead to the extinction of some important medicinal plant species.

The people of high-elevation territories rely on traditional therapies to treat different illnesses. The inhabitants of such a friable environment are greatly influenced by climatic, cultural, and biological variation. In Pakistan, the HKH (Himalaya-Karakoram and Hindukush) region contains a substantial rural population and diverse linguistic. These mountainous regions, extended in the extreme north of Pakistan are an extensive source of wood, timber forage, phytomedicines, etc. Evident influence on biodiversity, in response to seasonal variation, has been reported in terms of changes in the phenology of species and their altered distribution range [9]. Some species are either thriving or adapting in response to the changing environment [13,14]

The biodiversity of conservation parks in Pakistan has a much-known medicinal history. For instance, *Artemisia* belongs to the family *Asteraceae* and was acclaimed for having antimalarial properties and being effective in wound healing [15,16] having bioactive compounds such as alkaloids and Coumarin derivatives, their emission is not consistent throughout fall and summer such as the greatest number of compounds is emitted in July, followed by September and April [17,18]. Multiple ethnobotanical surveys documented the rich biodiversity of the KNP region comprised of 43 potential medicinal plant species belonging to 28 families [19]. In abundance, *Asteraceae, Fabaceae, Lamiaceae, Rosaceae, Chenopodiaceae, and Elaeagnaceae* were significantly observed. These potential plants are of higher importance as they are sources of biomolecules and various nutraceuticals [20]. It has been reported that the accumulation of phenylpropanoids is highly affected by temperature and precipitation variation [21]. Cold stress increased phenolic production in cereals and their subsequent incorporation into the cell wall [22]. Stresses such as both warm and cold water affect the flavonoids of *Pinus pinaster*s [23].

Furthermore, in aerial parts of *Ginkgo biloba*, temperature increases the production of terpenes and fewer phenolics [24]. The growth pattern of *Melastoma malabathricum* is widely influenced by lower temperatures, as they grew better and produced a higher amount of anthocyanin at 20 ± 2 °C [25]. Cold stress given to the Datura flower increased serotonin levels [26]. The concentration of phenolic compounds (e.g., flavonoids and tannins) decreases with high temperature, in contrast, the concentration of terpenoids increases [27]. However, fluctuation in allelochemicals does vary among species [28].

Considering the need to study the adaptability of high-elevation medicinal plants, the study aimed to establish an in-field manipulative experimentation model for evaluating the adaptation of selected medicinal plants through biochemical analysis. This research measured the worth of phytochemically significant plants in producing bioactive compounds under varying environmental conditions that will help preserve the ecological balance in the future.

## Methods

### Study design

**Fig 1:**
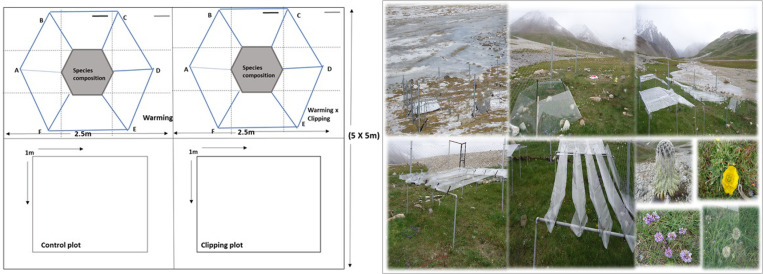
An example enclosure with open-top warming chambers (OTCs) and control plots is shown on the left. One such enclosure is shown in the image on the right, where the OTCs can be seen in the center of the picture. control plots are located in the space between the clipping plots and the OTCs [30].

In the Khunjerab National Park (KNP), a multisite manipulative experimental setup was established to induce climate warming. The study area is situated in the HKH (Hindukush-Karakoram-Himalaya) mountain ranges close to the Chinese border (36.37°N, 74.41°E), and covers an area of 4,455 km^2^. Across different vegetation zones, the five separate manipulative experiments were installed along the elevation gradient ranging from 3,590m to 4,696m. The experiments were designed using a randomized block design (RBD). Each site had a 5 x 5 m fenced area separated into two 2.5 x 2.5 m subplots. The five chosen sites were on generally level terrain, reflected the broader landscape, and featured a variety of shrubs and herbs, including *Astragulus, Potentilla, Artemisia and Primula,* etc. In the enclosure, two subplots were subjected to a heating treatment using six-sided open-top chambers that increase the temperature from of 1 to 1.5 °C [29] as compared to control plot (Fig 1). The fieldwork for this study was conducted under the Snow Leopard Foundation’s funded project titled “Multisite Experimentation on Rangeland and Grassland Ecosystems (MERGE).” Appropriate permits for this work were obtained from the Gilgit-Baltistan Parks and Wildlife Department, which approved access to the field sites through authorities.

### Sampling of plant species

For the biochemical analysis, *Astragulus peduncular* (AP), *Potentila melaleuca* (PM), *Artemisia rupestris* (AR), *Plantago major* (PM), *Primula macrophylla* (PrM), and *Poa alpina* (PA) plant species were selected based on the therapeutic potential [30]. These species were sampled during their peak growing season that starts from late March and last till late August [31,32].The plants that responded positively to the warming treatment were selected for further analysis. They were clipped 1 cm above ground, freeze-dried, and stored until further investigation. The research was conducted under the ethical oversight of an ethics committee and received prior review and approval from the Institutional Ethics Review Board (approval no. CIIT/BIO/ERB/17/53) and the Institutional Biosafety Committee (approval no. CIIT/BIO/ERB/17/04) at COMSATS University Islamabad, formerly known as COMSATS Institute of Information Technology (CIIT) Islamabad.

### Preparation of plant methanol extracts

Alcoholic solvents tend to extract the richest flavonoid content [33]. The extraction method described by [2] was adapted with 80% methanol as the solvent. Dried samples were ground with liquid nitrogen and kept at room temperature. 5 mL of methanol was added to each sample, transferred to 15 mL Falcon tubes, filtered through 0.45 μm filter paper, and stored at -80°C for future analysis.

### Phytochemical analysis

#### Total phenolic content (TPC).

A 500 mL aliquot of an extract diluted to 1:10 was mixed with 1 mL of Folin-Ciocalteu reagent, which had been further diluted to 1:100 using deionized water. This mixture was incubated in the dark for 10 minutes. Then, 1 mL of 7.5% Na_2_CO_3_ solution was added, and the mixture was centrifuged to remove excess Na_2_CO_3_. Absorbance measurements were recorded at 760 nm. Total phenolic content (TPC) was determined using a standard curve based on gallic acid concentrations of 25, 50, 75, and 100 mg/mL. The results are expressed as milligrams of gallic acid equivalents per 100 grams of dry weight (mg GAE/100 g DW). All measurements were conducted in triplicate for accuracy.

#### Total flavonoid content (TFC).

A modified AlCl_3_ colorimetric assay assessed plant species’ total flavonoid content (TFC). 1 mL of sample extract was combined with 1 mL of 5% AlCl_3_ solution in methanol; absorbance measurements were then performed three times using a spectrophotometer. A quercetin calibration curve, prepared by dissolving 5 mg of quercetin into 1 mL of methanol before serially diluting this solution down until 0.25 mg QE/100 gDW) was then calculated from its regression equation and reported as TFC for plant species evaluated [34].

#### High-performance liquid chromatography method (HPLC).

Methanolic fractions from crude extracts of *A. penduncularis*, *P. hololeuca*, *A. rupestris*, *P. major*, *P. macrophylla*, and *P. alpina* were analyzed for phenolic acids and flavonoids concentration using the method described by Hussain et al. (2013) [35]. Standards and mobile phase solvents were sourced from Sigma-Aldrich (United States). The analysis of phenolic compounds was performed using an LC-10A system equipped with a C18 column (dimensions: 250 × 4.6 mm, pore size: 5 µm, Phenomenex, Jupiter 5u C18, 300A). A UV-Vis detector was utilized with LC-10 software (Shimadzu RF-530, fluorescence monitor), and the column temperature was maintained at 30°C.Flavonoid standards, including rutin, quercetin, myricetin, kaempferol, and catechin, were prepared at a concentration of 10 µg/ml in acetonitrile. For phenolic acid standards, including syringic acid, p-hydroxybenzoic acid, vanillic acid, ferulic acid, Gallic acid, chlorogenic acid, p-coumaric acid, caffeic acid, and sinapic acid, the concentration was set at 20 µg/ml in methanol. The mobile phase for flavonoid identification consisted of acetonitrile and methanol (80:20) with 3% acetic acid, while the mobile phase for phenolic acids was composed of acetonitrile and distilled water (10:88) with 2% acetic acid. These mobile phases were filtered using a 0.45 µm × 47 mm filtration assembly and sonicated for 10 minutes (Model-EYELA, Toyota). Prior to applying the standards, the column was purged to eliminate any residual mobile phase. The solvent flow rate and maximum pressure were set at 1 ml/min and 250 kgf/cm², respectively. A volume of 20 µl from each standard was injected into the microinjector, and the retention times were recorded for reference. Peaks detected by the UV detector were analyzed, and the peak areas were calculated to quantify the phenolic compounds in the methanolic fractions.

### Data analysis

Analytical curves were established for TPC, TFC, and HPLC to measure each compound’s concentrations. Calibration curves for flavonoids and phenolic acids were created with standard concentrations (5, 10, 15, 20, and 25 µg/ml for flavonoids; 10, 20, 30, 40, and 50 µg/ml for phenolic acids). Linear regression was used to obtain excellent correlation coefficients (R² > 0.99) for precise quantification. To ensure uniformity, each calibration curve was conducted under identical conditions as the samples. The clarity of chromatographic peaks was closely examined to avoid overlapping or misidentification of substances. To guarantee accuracy, retention periods for each standard were recorded and compared to peak values from the samples. The chromatographic technique provided adequate peak resolution for all targeted substances, with resolution values greater than 1.5 for neighboring peaks. Potential challenges included co-elution of compounds and baseline noise, mitigated by optimizing the mobile phase composition and using a thermostatically controlled column to ensure consistent separation. Any ambiguous peaks were excluded from the analysis to maintain the reliability of the results. Data presented are mean ± standard deviation values obtained through triplicate experiments. To evaluate differences between warming and control plots, ANOVA was employed with a significance level of p < 0.05 followed by Tukey’s multiple comparison tests to detect differences among treatments Pearson correlation was then utilized to examine any associations among variables. All statistical analyses were completed on R software version 3.5.1 [36]. Bar plots were generated using *tidyverse* and *ggplot* packages in R while species abbreviations are as follows: AP = *Astragalus penduncularis,* AR = *Artemisia rupestris* PA = *Poa alpina,*PH = *Potentilla hololeuca* PM = *Plantago major* PrM = *Primula macrophylla.*

## Results

### Influence of climate variation on secondary metabolic profile of important medicinal plant species in manipulative experiments

The selected species for metabolic profiling demonstrated potential therapeutic properties, allowing for the evaluation of secondary metabolic characteristics and the assessment of any changes in the concentration of key phenolic and flavonoid compounds due to warming treatment [30].

**Fig 2:**
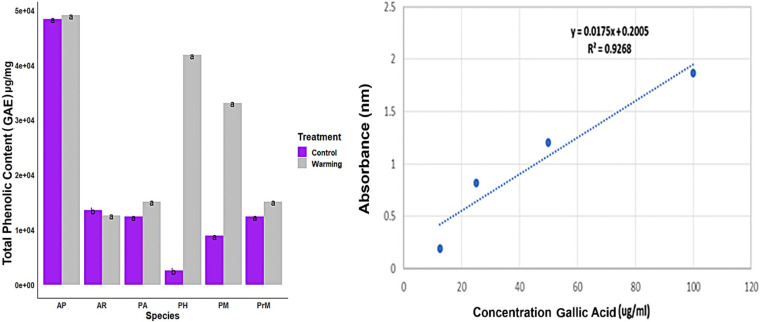
Distribution and concentration of total phenolic content (TPC) across species in warmed versus control plots, expressed as milligrams of Gallic acid equivalents per gram of dry weight, using triplicate measurements; data points show mean ± standard error from triplicate measurements while columns with letters representing statistically significant differences (p < 0.05).

The statistical analysis showed a significant variation among the species investigated in control and warming plots. These findings showed that extracts have the highest TPC in the warming plots for *A. penduncularis* (AP) the TPC content in the warming plot was 4035.016 (μg/ml) GAE/g and in control plots, it was 5324.842 μg/ml GAE/g though this difference is not significant but apparent as shown in (Fig 2). Meanwhile, species like *A. rupestris* (AR) and *P. hololeuca* (PH) showed significant differences in TPC in control and warming plots. *P. hololeuca* had the lowest accumulation of TPC (in control plots whereas in warming plots it accumulated more phenolic compounds. We performed analysis of variance (ANOVA) and Tukey HSD to identify which plant species significantly differ in accumulation of TPC within and between the treatments (p < 0.001). However, all the plant species show higher TPC in warming plots.

**Fig 3:**
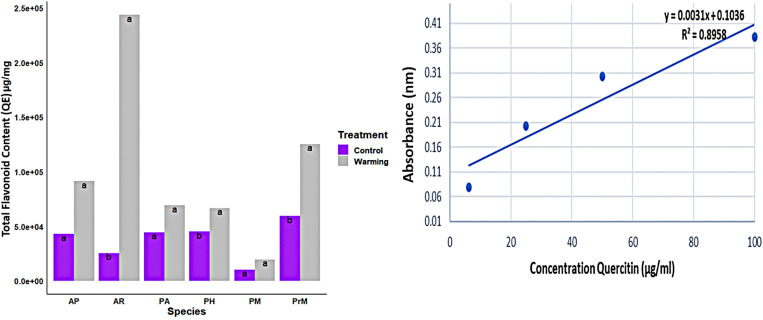
TFC for each species from warmed and control plots, was calculated, and expressed as milligrams of quercetin equivalents per gram of dry weight. Significant differences (p < 0.05) were assessed bar plots created using R packages like tidyverse and ggplot packages.

The analysis of TFC among treatment *vs* control revealed significant differences among species and in treatment plots. These results were statistically validated using ANOVA (p < 0.001) combined with Tukey test (Fig 3). *A. rupestris* AR and *P. macrophylla* PrM had the highest TFC in warming plots, with 209.8 mg quercitin/g, and 100.5 mg quercitin/g respectively. If the treatment vs control was focused there were statistical differences in TFC in other species like *P. alpina* and *P. hololeuca* showed highly significant variation in TFC content in treatment plots (Fig 3). TFC levels of *P. major* PM and *A.peduncularis* AS did not vary significantly in control and warming treatment though the concentration was lower in control plots.

### Determination and quantification of some phenolic compounds under warming treatment by HPLC

**Fig 4:**
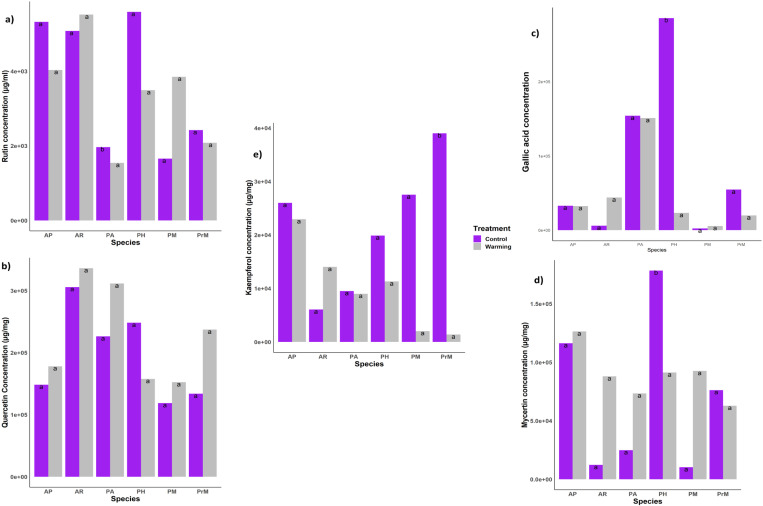
(a)–(e) illustrates variations in phenolic compounds between warmed and control plots, quantifying concentrations of rutin, quercetin, Gallic acid, myricetin and kaempferol within selected medicinal species and presented as mean + standard error data points with columns labeled with various letters to denote significant differences (determined using Tukey’s honestly significant difference test). Figures were created with tidyverse, scales and ggplot2 packages available within R.

HPLC analysis of extracts from various plant species produced retention times that corresponded with nine specific compounds, providing evidence of climate variation by measuring the concentration of each compound within species (Fig 4a–e).

**Fig 5:**
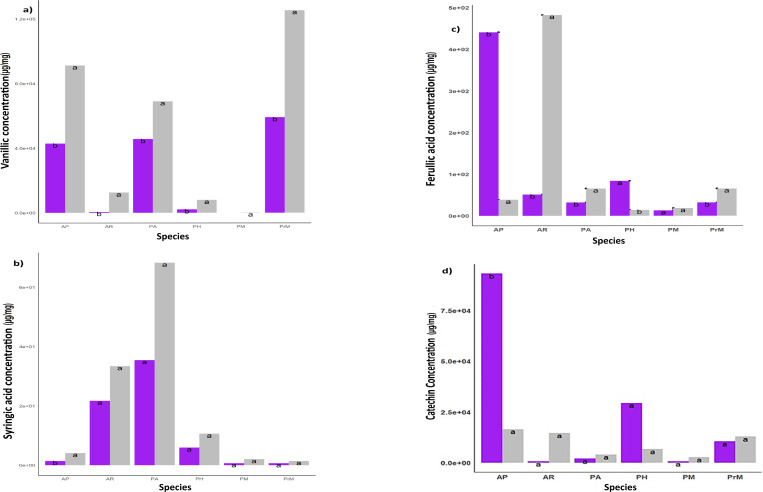
(a)–(d) Phenolic compounds among warming and control plots. The minor compounds concentration in all selected medicinal species. Each data point was present with the average means ± SE.

Analysis showed a vast range of variation between warming treatment plots as compared to the control plot. Where there was a difference in phenolic compound concentration, it was found that each plant species responded differently towards warming either by increasing or decreasing the accumulation of phenolic compounds. Fig 4 represents this variation in the phenolic compound content among species and warming vs control (p < 0.01) suggesting the presence of major (Fig 4a–e) and minor (Fig 5a–d) compounds. The major identified compounds were Gallic acid (157.29 µg/ml), quercitin 237.11 µg/ml, rutin (159.22 µg/ml), kaempferol (157.29 µg/ml), and myricetin (79.83µg/ml) (Fig 3). The average concentration of these compounds in plant species is given in S1–S4 Tables.

The identified compounds were classified according to the concentration of specific phenolic acids in control and warming treatment. The accumulation of these compounds showed the individualistic response of species under warming stress and explained their potential for survival in times of ongoing climate warming. The major compounds were rutin, quercetin, Gallic acid, myricetin, and kaempferol and minor compounds include syringic acid, vanillic acid, catechin, and ferulic acid (Fig 5a–d).

The identified minor compounds (Fig 5) include ferulic acid (109.82 µg/ml), syringic acid (12.69 µg/ml), vanillic acid (2.297 µg/ml), and catechin (13.79 µg/ml), their concentration was less than the major compounds (S5–S8 Tables). The variability among plant species and within treatment is highlighted by the presence of each compound in plant species and their accelerated concentration in response to warming. There was variation in the content of minor compounds as well such as vanillic acid (2.29 µg/ml) produced in very small quantities in all species except *P. macrophylla* (Fig 5a). The concentration of syringic acid was less in all plant species except in *P. alpina* (68.2 µg/ml). Also, ferulic acid concentration was high in control plots of *A. penduncularis* (440.8 µg/ml) as compared to the other plant species that produced ferulic acid in minute quantity (S5–S8 Tables). However, the high concentration of major compounds accumulated in all species (Fig 4a–e) but under warming, the rate of accumulation was significantly high suggesting more production of active compounds under a stressed environment.

**Fig 6:**
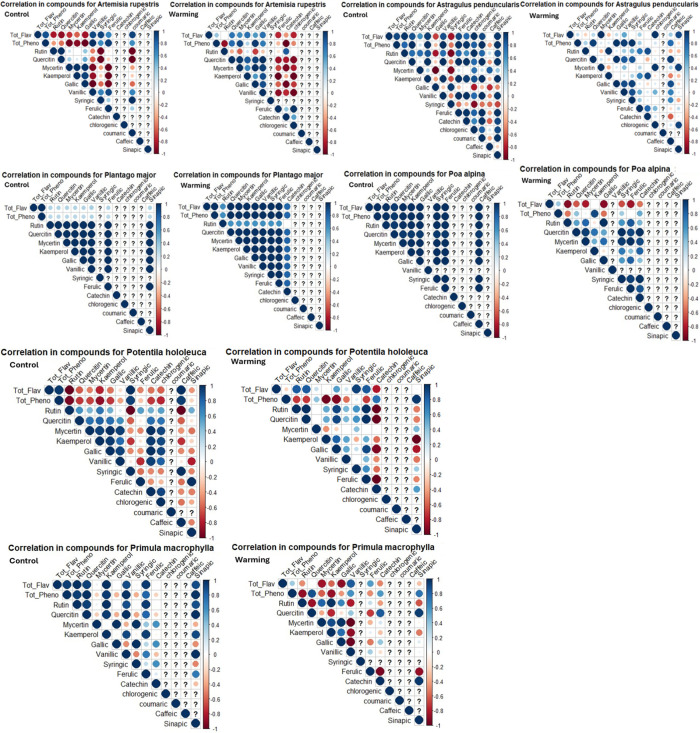
Pearson’s correlation between active plant compounds and their total phenolic and total flavonoid contents in warming and control experiments, where circles with increasing intensity represent increasing correlation coefficient strength while their size corresponds to the strength of correlation coefficients; on the right side of this correlogram is also displayed their values and colors to them using ggcorrplot package visualization software.

Moreover, the association between total phenolic content, total flavonoid content, and major and minor phenolic compounds is shown in the selected parameters among all species (Fig 6). There were positive correlations +[6] between rutin, quercetin, myricetin, and kaempferol in control and warming plots of plant species.

## Discussion

Plant secondary metabolite concentration and accumulation play a significant role in determining its ecological interaction and contribute to predicting the future dynamics of an ecosystem. High mountainous medicinal plant species respond positively to the OTC-induced warming by increasing their biomass and percent cover [36].These finding suggest that increasing temperature will favor the growth of some species at higher altitude grasslands, but at the same time, it will decrease the biodiversity [29]. Considering these findings, the results of this study indicated that the plant species’ defense mechanism is also affected by climate change. Plant secondary metabolism fluctuates in response to increasing temperature [37–39]. This prediction is based on changes in the concentration of key metabolites [40]. The highest phenolic content was found in *A. penduncularis, P. hololeuca*, and *P. major*, while the richest among them was *A. penduncularis*.The concentration of flavonoids in *A. penduncularis* did not show any significant difference in warmed plots. Similarly, among different species, TPC varied even in control plots (Fig 3), dispensing the biological understanding of plant secondary metabolic regulation by linking the secondary metabolites’ responses and growth and defense mechanism.[37] Temperature is the main driver of the structure and functioning of fragile ecosystems such as HKH. [38] It has been reported that the rich biodiversity of this region, designated is at higher risk[39] as shown by the study results, in response to warming, some species will outcompete others leading to loss of biodiversity. Not only are higher elevations at risk, but species migration behavior has been observed at lower elevations too[29] This invasion of new species at higher elevations will leave no habitat for the native species as they will outcompete, so they will migrate to even higher elevations not suitable for their growth exposing their metabolic profiles to further stress[40,41]. So, it is predicted that the accumulation of the phenolic compounds under warming will change the underlying defense mechanisms.[42,43] It has been shown that plants under any stress fix more carbon dioxide consumed by secondary metabolites thus the production of phenolic compounds increases. [44] However many species showed variation in accumulation of TPC and TFC such as chemical composition of essential oil of Mentha species showed high phenolic content at low temperatures (5% in winters). The increase in winter temperature with a long day photoperiod increases carbon assimilation and aboveground biomass production[43,44] The seasonal variation in the composition of essential oil was found in the early summer and early spring, explaining the fact that long photoperiods increased the phenolic monoterpenoids in the oil). There is no such compiled information representing optimized season for an accumulation of pharmaceutically important constituents from medicinal plants. Some studies reported that elevated temperatures induce a high concentration of volatile organic compounds [45]. In such stress conditions, the downregulation or upregulation of genes affects the growth of plants and causes modifications in biochemical processes, enabling them to develop a high tolerance to high temperatures. Such tolerance is species-specific, as shown in our study; out of 17 species, six species developed tolerance to high temperatures and showed a high accumulation of TPC and TFC. In the absence of grazing, warming enhanced their cover and biomass production, such as *A. rupestris* and *P. hololeuca*, the stress-induced high accumulation of TFC (Fig 3). The secondary chemicals are found to be species-specific and chemical-specific, too, as plant chemical responses induced by climate factors are highly variable. However, some pattern specificity of chemicals in response to different environmental stressors has been found [43,46]. For example, both elevated temperature and increased levels of CO_2_ [47–49]enhance phenolic acid concentration, but it is found that flavonoid accumulation is also increased [44,50] As in the signaling processes, flavonoids promote symbiosis between plants and bacteria by nodule formation [51]So, they intervene in plant environment interaction. Therefore, a high concentration of TFC is found in plant species as part of their adaptive strategy [52]. These findings strengthen the results of metabolic analysis by the HPLC analysis (HPLC analysis identified that major phenolic compounds concentration was increased, similar studies have shown the temperature extremes and effect of plant growth which is associated with accumulation of these metabolic and therapeutic compounds [39,42]. Major compounds that showed the fluctuation in warm vs control points were Gallic acid, rutin quercetin, myricetin, and kaempferol (Fig 4). Rutin, an antioxidant and anti-inflammatory flavonoid, showed high accumulation in *A. peduncular (AP)*, *A. rupestris (AR)*, *P. macrophylla*, *P. major*, and *P. hololeuca* (PH). Notably, the concentration of rutin in *A. penduncularis* did not show any significant difference in warmed plots as compared to control ones, indicating its stability under temperature-stress conditions [53,54]. Similar studies have shown that rutin concentration in asparagus remains stable and doesn’t vary significantly between high and low temperatures (4─20 °C) [55]. This stability of rutin suggests that these medicinal plant species will maintain their anti-inflammatory and antioxidant potential under high-elevation warming conditions [53]. However, *P. alpina* showed a high variation in concentration of rutin between the control and warming plot, which restate the species-specific behavior and adaptation as a defense mechanism [55,56]. In the accumulation of quercetin, all species showed non-significant variation in the concentration (Fig 4b).

Minor compounds such as *Gallic acid, Ferulic acid, Syringic acid*, and *Vanillic acid* show highly specific and varying accumulation levels in control and warming plots. The varying concentrations of these compounds in all species reinforce their immense importance in understanding and managing plant-environment interactions [29,38,57].

Recent research has demonstrated the metabolism of Gallic acid fluctuated in response to drought stress in oaks [44,58]. These findings are consistent with the outcomes of this study, where low levels of Gallic acid were found in the *P. hololeuca* under warming treatment explaining the metabolism dynamics [39].

Syringic and Ferulic acid have high pharmaceutical potential to modulate enzyme activities involved in blood sugar and cancer [59]. The findings of this study reveal that *A. rupestris* and *P. alpina* had a high concentration of these compounds, which represents the high medicinal importance of these plant species, as explained by local informants in an ethnobotanical survey[36]. Moreover, the high value of TPC and TFC indicates the high antioxidant and anti-inflammatory activities of these plant species and illuminates the reveals potential utility of these medicinal plants in therapeutic context [12,60].

Temperature, elevation, and other related factors, such as soil moisture and sunlight, play a constitutive role in secondary metabolic accumulation [36,46,61]. Our findings suggest that differences in the distribution and accumulation of phenolic compounds in different species can be attributed to environmental stress and interaction and cannot relate to the geographic distance [62]. Thus at different altitudes, their functional bioactive compounds drive the adaptation of endemic medicinal plants to environmental conditions [63,64]. To survive, these species are highly dependent on the increasing activity of their metabolic enzymes to adapt to high-altitude changing conditions [65]. A great variation in the concentration of secondary metabolites among species will possibly lead to a prominent difference in their medicinal efficacy [43]. In light of our findings established as baseline knowledge, it is suggested to observe the long-term impacts of climate variables and their synergistic effects on plant therapeutic potential including the evolution of plant adaptation patterns, concentration of secondary metabolites, and influence of warming on metabolic pathways, migration from low to higher elevation and extinction vulnerabilities of such grassland and rangeland ecosystems [66].

High-elevation plants opt to survive by exhibiting certain traits such as short height, less cover, and denser inflorescence, such adaptation improves their biomass production and survival rate [39,67]. Many studies have explored the abiotic stress tolerance in different plant biochemical profiles, but insufficient attention is given to the culturally and medicinally significant flora of the HKH region. Current research addresses this gap by examining the biochemical and metabolic profiles of native plants thus revealing their complex interplay between adaptation strategies and ecological factors. Other molecular studies illustrate the role of genes in altitude-dependent trait selection. Various theories explain the mountain biodiversity through different experimentation models by geological shift and rangeland dynamics of alpine systems [68,69]. In our study, we brought novel insights by introducing open-top chambers in high-elevation environments to simulate climate warming. This is the first study examining secondary metabolic profiles of medicinal plants, such as specific species, under OTC-induced warming in the Himalayas, Karakoram, and Hindukush regions It offers a controlled system to examine biochemical responses and showcases the metabolic adaptations and biochemical responses. Previous studies have used OTC experiments to analyze metabolic profiles of aromatic plants under elevated temperature conditions and propose the potential of that could alter the accumulation of primary and secondary metabolic profiles and their therapeutic properties [69–71]. Similarly, emphasis on complex biotic response to warming was reported by documenting the changes in chemical traits of *Aristolochia chilensis* [72]underscoring the significant performance of OTCs to maximize interpretation of plant responses [69,73,74]. Contrary to previous reports on ecosystems, progressive vegetative growth was observed in selected medicinal plants such as Artemisia, Plantago major, Astragulus, etc. suggesting the distinct potential of adaptations depends upon plant functional traits and specific defense system which is crucial in determining the future of these plants under changing climate conditions [39,73–75].

## Supporting information

S1 Table Effect of warming treatment on the accumulation of Rutin. (DOCX)

S2 Table Effect of warming treatment on the accumulation of Myricetin. (DOCX)

S3 Table Effect of warming treatment on the accumulation of Kaempferol. (DOCX)

S4 Table Effect of warming treatment on the accumulation of Gallic acid. (DOCX)

S5 Table Effect of warming treatment on the accumulation of Vanillic acid. (DOCX)

S6 Table Effect of warming treatment on the accumulation of Syringic acid. (DOCX)

S7 Table Effect of warming treatment on the accumulation of Ferulic acid. (DOCX)

S8 Table Effect of warming treatment on the accumulation of Catechin. (DOCX)
