# Supplementary material for: Influence of open-top chambers induced climate warming on secondary metabolic profile of culturally and medicinally important plants of Himalaya, Karakoram and Hindukush
Source: PLoS One. 2025 May 14;20(5):e0322480. doi: 10.1371/journal.pone.0322480 (PMC12077716; doi:10.1371/journal.pone.0322480)
Supplement: S1 Table — (DOCX) [file pone.0322480.s001.docx]

**Table S1. Effect of warming treatment on the accumulation of Rutin**

| *Plant species* | Control means | Warming mean | F-value | P-value |
| --- | --- | --- | --- | --- |
| *Astragulus penduncularis (AS)* | 5324.842 a | 4035.016 a | 0.66 | 0.428 |
| *Artemisia rupestris (AR)* | 5081.353 a | 5520.654 a | 0.362 | 0.556 |
| *Poa alpina (PA)* | 1965.739 b | 150042.877 a | 5.217 | 0.0364 * |
| *Potentila hololeuca(PT)* | 5591.442 a | 3493.071 a | 3.047 | 0.1 |
| *Plantago major (PM)* | 1657.614 a | 3850.761 a | 0.452 | 0.511 |
| *Primula macrophylla (PrM)* | 2420.924 a | 2081.473 a | 0.223 | 0.643 |
