# Supplementary material for: Influence of open-top chambers induced climate warming on secondary metabolic profile of culturally and medicinally important plants of Himalaya, Karakoram and Hindukush
Source: PLoS One. 2025 May 14;20(5):e0322480. doi: 10.1371/journal.pone.0322480 (PMC12077716; doi:10.1371/journal.pone.0322480)
Supplement: S3 Table — (DOCX) [file pone.0322480.s003.docx]

**Table S3. Effect of warming treatment on the accumulation of Kaempferol**

| *Kaempferol* |  |  |  |  |
| --- | --- | --- | --- | --- |
| *Plant species* | **Control means (Conc. ng/ml)** | **Warming mean** | **F-value** | **P-value** |
| *Astragulus penduncularis (AS)* | 26002.83 a | 22938.61 a | 0.051 | 0.824 |
| *Artemisia rupestris (AR)* | 6058.926 a | 14021.159 a | 4.155 | 0.0584 |
| *Poa alpina (PA)* | 9502.481 a | ---- | 0.008 | 0.929 |
| *Potentila hololeuca(PT)* | 19885.85 a | 11297.39 a | 2.364 | 0.144 |
| *Plantago major (PM)* | 27534.65 a | 2021.94 a | 3.416 | 0.0831 |
| *Primula macrophylla (PrM)* | 39009.987 a | 1383.342 b | 9.842 | 0.00636 ** |
