# Supplementary material for: Influence of open-top chambers induced climate warming on secondary metabolic profile of culturally and medicinally important plants of Himalaya, Karakoram and Hindukush
Source: PLoS One. 2025 May 14;20(5):e0322480. doi: 10.1371/journal.pone.0322480 (PMC12077716; doi:10.1371/journal.pone.0322480)
Supplement: S4 Table — (DOCX) [file pone.0322480.s004.docx]

**Table S4**. **Effect of warming treatment on the accumulation of Gallic acid**

| *Gallic acid* |  |  |  |  |
| --- | --- | --- | --- | --- |
| *Plant species* | **Control mean** | **Warming mean** | **F-value** | **P-value** |
| *Astragulus penduncularis (AS)* | 130103.04 a | 62509.03 a | 2.435 | 0.138 |
| *Artemisia rupestris (AR)* | 84912.15 b | 241023.76 a | 6.232 | 0.0238 * |
| *Poa alpina (PA)* | 15429.3 a | 151051.6 a | 3.973 | 0.0636 |
| *Potentila hololeuca(PT)* | 315519.50 a | 23222.02 b | 4.667 | 0.0463 * |
| *Plantago major (PM)* | 2279.690 a | 5624.304 a | 1.215 | 0.287 |
| *Primula macrophylla (PrM)* | 54748.70 a | 19856.07 a | 1.839 | 0.194 |
