# Supplementary material for: Influence of open-top chambers induced climate warming on secondary metabolic profile of culturally and medicinally important plants of Himalaya, Karakoram and Hindukush
Source: PLoS One. 2025 May 14;20(5):e0322480. doi: 10.1371/journal.pone.0322480 (PMC12077716; doi:10.1371/journal.pone.0322480)
Supplement: S5 Table — (DOCX) [file pone.0322480.s005.docx]

**Table S5**. **Effect of warming treatment on the accumulation of Vanillic acid**

| *Vanillic acid* |  |  |  |  |
| --- | --- | --- | --- | --- |
| Plant species | **Control mean** | **Warming mean** | **F value** | **P value** |
| *Astragulus penduncularis (AS)* | 42835.56 b | 91197.78 a | 12.49 | 0.00275 ** |
| *Artemisia rupestris (AR)* | 517.1667 b | 12569.2944 a | 6.187 | 0.0243 * |
| *Poa alpina(PA)* | 45664.33 b | 69037.78 a |  |  |
| *Potentila hololeuca(PT)* | 2122.067 b | 7995.883 a | 11.51 | 0.00372 ** |
| *Plantago major (PM)* | 5.477778 a | 192.305556 a | 3.772 | 0.0699 . |
| *Primula macrophylla(PrM)* | 59144.44 b | 125255.56 a | 42.11 7 | .47e-06 *** |
