# Supplementary material for: Influence of open-top chambers induced climate warming on secondary metabolic profile of culturally and medicinally important plants of Himalaya, Karakoram and Hindukush
Source: PLoS One. 2025 May 14;20(5):e0322480. doi: 10.1371/journal.pone.0322480 (PMC12077716; doi:10.1371/journal.pone.0322480)
Supplement: S6 Table — (DOCX) [file pone.0322480.s006.docx]

**Table S6**. **Effect of warming treatment on the accumulation of Syringic acid**

| *Syringic acid* |  |  |  |  |
| --- | --- | --- | --- | --- |
| Plant species | **Control mean** | **Warming mean** | **F value** | **P value** |
| *Astragulus penduncularis (AS)* | 1.400000 b | 4.108889 a | 4.577 | 0.0482 * |
| *Artemisia rupestris (AR)* | 21.71 a | 0.00 a | 4 | 0.0628 **.** |
| *Poa alpina(PA)* | 35.44444 a | 68.25556 a | 1.778 | 0.201 |
| *Potentila hololeuca(PT)* | 5.947778 a | 10.631111 a | 1.36 | 0.261 |
| *Plantago major (PM)* | 0.000000 a | 2.043333 a | 4 | 0.0628 . |
| *Primula macrophylla(PrM)* | 1.41 a | 0.00 a | 4 | 0.0628 . |

Signif. codes: 0 ‘***’ 0.001 ‘**’ 0.01 ‘*’ 0.05 ‘.’ 0.1 ‘ ’ 1
