# Supplementary material for: Influence of open-top chambers induced climate warming on secondary metabolic profile of culturally and medicinally important plants of Himalaya, Karakoram and Hindukush
Source: PLoS One. 2025 May 14;20(5):e0322480. doi: 10.1371/journal.pone.0322480 (PMC12077716; doi:10.1371/journal.pone.0322480)
Supplement: S7 Table — (DOCX) [file pone.0322480.s007.docx]

**Table S7**. **Effect of warming treatment on the accumulation of Ferulic acid**

| *Ferulic acid* |  |  |  |  |
| --- | --- | --- | --- | --- |
| Plant species | **Control mean** | **Warming mean** | **F value** | **P value** |
| *Astragulus penduncularis (AS)* | 440.86667 a | 38.65556 a | 3.916 | 0.0653 . |
| *Artemisia rupestris (AR)* | 51.25556 b | 482.33556 a | 23.6 | 0.000175 *** |
| *Poa alpina(PA)* | 32.03778 b | 65.62222 a | 15.53 | 0.00117 ** |
| *Potentila hololeuca(PT)* | 83.99222 a | 14.23556 b | 5.507 | 0.0321 * |
| *Plantago major (PM)* | 12.83778 a | 18.58667 a | 0.259 | 0.618 |
| *Primula macrophylla(PrM)* | 32.03778 b | 65.62222 a | 15.53 | 0.00117 ** |

Signif. codes: 0 ‘***’ 0.001 ‘**’ 0.01 ‘*’ 0.05 ‘.’ 0.1 ‘ ’ 1
