# Supplementary material for: Influence of open-top chambers induced climate warming on secondary metabolic profile of culturally and medicinally important plants of Himalaya, Karakoram and Hindukush
Source: PLoS One. 2025 May 14;20(5):e0322480. doi: 10.1371/journal.pone.0322480 (PMC12077716; doi:10.1371/journal.pone.0322480)
Supplement: S8 Table — (DOCX) [file pone.0322480.s008.docx]

**Table S8**. **Effect of warming treatment on the accumulation of Catechin**

| Catechin |  |  |  |  |
| --- | --- | --- | --- | --- |
| Plant species | **Control mean** | **Warming mean** | **F value** | **P value** |
| *Astragulus penduncularis (AS)* | 93058.50 a | 16406.48 b | 4.599 | 0.0477 * |
| *Artemisia rupestris (AR)* | 0.4557 b | 14507.19 a | 10.74 | 0.00475 ** |
| *Poa alpina(PA)* | 0. 12340 b | 3938.977 a | 15.65 | 0.00113 ** |
| *Potentila hololeuca(PT)* | 29335.793 a | 6646.519 b | 13.73 | 0.00192 ** |
| *Plantago major (PM)* | 0.57675 a | 2580.396 a | 2.588 | 0.127 |
| *Primula macrophylla(PrM)* | 10432.97 a | 12791.41 a | 0.059 | 0.811 |
